# Salinity tolerance mechanisms and their breeding implications

**DOI:** 10.1186/s43141-021-00274-4

**Published:** 2021-11-09

**Authors:** Mandeep Singh, Usha Nara, Antul Kumar, Anuj Choudhary, Hardeep Singh, Sittal Thapa

**Affiliations:** 1 Department of Plant Breeding and Genetics, Punjab Agricultural University, Ludhiana, Punjab 141004 India; 2 Department of Botany, Punjab Agricultural University, Ludhiana, Punjab 141004 India; 3 Department of Agronomy, Punjab Agricultural University, Ludhiana, Punjab 141004 India

**Keywords:** Salinity tolerance, Ion homeostasis, Novel biotechnological approaches, GWAS, Heat shock proteins

## Abstract

**Background:**

The era of first green revolution brought about by the application of chemical fertilizers surely led to the explosion of food grains, but left behind the notable problem of salinity. Continuous application of these fertilizers coupled with fertilizer-responsive crops make the country self-reliant, but continuous deposition of these led to altered the water potential and thus negatively affecting the proper plant functioning from germination to seed setting.

**Main body:**

Increased concentration of anion and cations and their accumulation and distribution cause cellular toxicity and ionic imbalance. Plants respond to salinity stress by any one of two mechanisms, *viz*., escape or tolerate, by either limiting their entry via root system or controlling their distribution and storage. However, the understanding of tolerance mechanism at the physiological, biochemical, and molecular levels will provide an insight for the identification of related genes and their introgression to make the crop more resilient against salinity stress.

**Short conclusion:**

Novel emerging approaches of plant breeding and biotechnologies such as genome-wide association studies, mutational breeding, marker-assisted breeding, double haploid production, hyperspectral imaging, and CRISPR/Cas serve as engineering tools for dissecting the in-depth physiological mechanisms. These techniques have well-established implications to understand plants’ adaptions to develop more tolerant varieties and lower the energy expenditure in response to stress and, constitutively fulfill the void that would have led to growth resistance and yield penalty.

## Background

Global food demand is continuously increasing, and with skyrocketing population, it is expected to double in the near future. Feeding the world population with the available limited natural resources is not an easy task [1–3]. Attributed to several biotic and abiotic stresses, genetic potential of the crops is not fully exploited. Abiotic stresses encompass raised salinity, temperature and drought, alleviation in soil oxygen, pollutants, high UV radiation, and inadequate mineral nutrients [3, 4]. Breeding approaches provide an insight for salinity stress tolerance as reported in some crops such as rice [5] and wheat [6]. Integration of comparative, functional, and structural genomics would boost the traditional breeding efforts. Genetic manipulation methods have been utilized in crop plants to recognize genes related to salt tolerance and their introgression [7].. Utilization of molecular tools in breeding programs is the most valuable upshot of biotechnology [8, 9]. However, an enormous space between the crop yields in stress conditions and optimal conditions is still leftover.

Salinity stress is a physiological outcome of excessive salt in plant cell which has detrimental effects on plant’s metabolism. Soils are stratified as saline when the ECe (Electrical Conductivity of a saturated soil Extract) is ≥ 4 dS/m which is roughly equivalent to 40 mM NaCl and approximately give rise to osmotic pressure of 0.2 megapascal (MPa) [10]. However, plants have numerous morphological (early flowering, prevention of lateral shoot development, and root adaptations), physiological responses (stomatal responses, osmotic adjustment, Na^+^/K^+^ discrimination, and ion homeostasis), and biochemical responses (antioxidant activity, polyamines, change in hormone level, increased proline level) under salinity stress, forming it as a complex phenomenon [11–13]. Abscisic acid (ABA) is a major phytohormone which plays a significant role in improving the performances of plants during stress conditions such as salinity, low temperature, and drought [14]. ABA production in the plants alleviates the effects of salinity on the growth, translocation of assimilates, and photosynthesis [15, 16]. Application of sodium nitroprusside (SNP) in soybean (*Glycine max L.*) enhances the physiological and morphological attributes under saline conditions. It has been reported that SNP as nitic oxide (NO) donor could greatly increase the salinity tolerance and regeneration potential by mimicking of plant hormone and signal molecule [17].

**Fig. 1:**
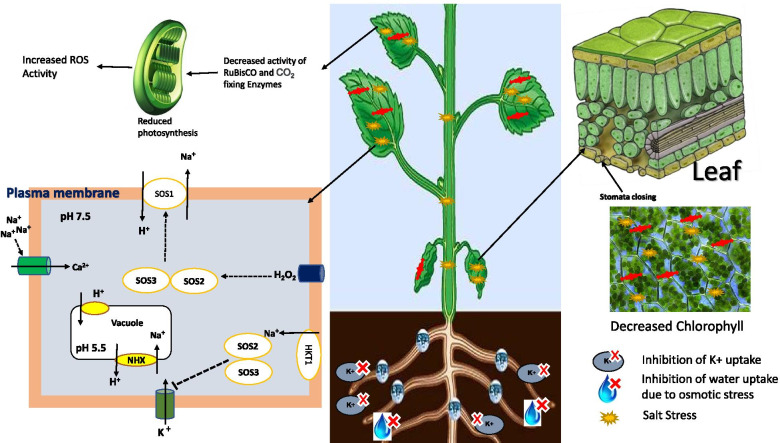
Dissecting mechanisms of salinity tolerance in plants

Soil salinity typically inhibits plant growth and reproduction through an initial osmotic stress phase followed by ionic toxicity due to accumulation of Na^+^ and Cl^−^ ions in the cell cytosol that results ultimately in oxidative stress and nutritional deprivation [18, 19]. Reactive oxygen species (ROS) scavenging, ion homeostasis, osmotic adjustments, and metabolic activities are greatly affected when plants are subjected to salinity stress. In order to overcome these various abiotic stresses, plants accumulate compatible harmless biomolecules which play a significant role in plant processes. These include polyamines [20, 21]; heat shock proteins (HSPs) [21, 22]; nitric oxide (NO) [23, 24]; and hormones like ABA [14, 25], salicylic acid [26], and brassinosteroids [26, 27]. Late embryogenesis abundant (LEA) protein is found to be effective against various stresses such as cold, drought, and salinity [28, 29]. These accumulated solutes have a role in protein solubilisation like glycine, ectoine, and betaine and in uncharged solutes such as pinitol and mannitol which have a scavenging activity of ROS [21]. NO is a vaporous molecule having a significant role in the regulation of several developmental processes (cell death, seed germination, stomata closure, root growth, flowering, and respiration), plant growth, and response to stress and a role in signal transduction [23] (Fig. 1).

Various traits have been identified in several studies for which expression or presence connecting the plant adaptability to salinity stress conditions (Table 1). Genomic approaches and crop physiology provide new insights to breeders to overcome salinity stress using new emerging tools for crop improvement [9]. Plants cope with salinity stress using various mechanisms, and these mechanisms can be exploited using strategies as mentioned above. This paper emphasized salinity stress, its effect on plants, and their adaptive mechanisms and discussed new cutting-edge tools to cope with salinity stress in this era where food security is the major challenge.

**Table 1:** Biochemical and physiological responses of plants to salinity stress

Sr. no.	Plant traits	Yield-related impacts on plant	Variation in stress	References
1	Plants root growth	Inhibition of nutrients and water absorption	Stress lowers the osmotic potential of plant roots.	[30]
2	Leaf tissues	Necrosis and chlorosis	Salt in the cells produce toxicity, and antioxidant helps in lowering the toxicity.	[30]
3	Leaf anatomy	Impact on leaf tissue	Reduction in the epidermis and mesophyll thickness as well as decrease in the intercellular spaces	[31]
4	Oxidative damage	Cellular toxicity due to production of reactive oxygen species (ROS)	Plants having antioxidant activity tolerate this oxidative damage.	[31]
5	Osmotic potential	Accumulation of salt in the leaves cause injury to the leaves and roots of the plants.	Halophytes tolerate the salt stress by accumulation of salt in the leaves by modifying the osmotic potential but not glycophytes as they are less tolerant to salt.	[32]
6	Photosynthesis and photosynthetic pigments	Reduced photosynthetic capacity	Closing of the stomata by subjection of plant to salt for a short time increases the tolerance of plant to salt stress.	[33]
7	Gaseous change characteristics	Salt stress notably decreased the few gaseous changes characteristics like water use efficiency, transpiration rate, etc. in some cultivars of sunflower.	Salt concentration improves the chlorophyll ratio a/b as the amount of chlorophyll b may be transformed into chlorophyll a in the course of the process of degradation resulting in the increased concentration of chlorophyll a.	[32–34]
8	Reproductive development	Salinity caused sterility in some plants.	In response to salinity, plants modify itself by inducing early flowering and prevention of lateral shoot development.	[35]
9	Hormones	Increased concentration of ABA	Enhanced amount of ABA during salt stress attenuates the repressive effect of salinity on growth as well as translocation of assimilates.	[15, 36]
10	Amino acids	Decrease in the concentration of amino acids such as methionine, arginine, and cystine.	Increase in the amount of proline in response to salt stress	[37]
11	Carbohydrates	Agglomeration of trehalose, fructose, glucose, fructans, and starch.	In the carbon storage, osmoprotection and scavenging of ROS, these carbohydrates play a role in salt stress conditions.	[31, 38]

## Main text

### Physiological aspects of salinity tolerance

Tolerance to salinity is the plant’s potential to grow and flourish its life cycle in high saline conditions [11]. Salinity tolerance in crops varied according to the crop type. For example, barley is more tolerant to salt than rice, legumes are severe sensitive than cereals, and adequate salinity tolerance is there in case of Lucerne and Alfalfa [39]. The most deleterious effect imposed by salinity stress is ion toxicity. Ebrahim et al. [40] demonstrated that tolerance mechanisms such as ion exclusion (Na) present in the wild barley (*Hordeum vulgare*) cause much lower accumulation of Na in the root and shoot and better K/Na discrimination than in the cultivated barley, resulting in the higher survival rate under 300 mM NaCl for 4 weeks. When two chickpea varieties (Rupali and Genesis 336) subjected to salt stress were compared, they exhibited alleviated sugar content and presence of inositol, galactitol, mannitol, arabitol, xylitol, and erythritol, suggesting their roles under salt stress. In another study, inositol and sucrose were found to be highly accumulated in *Atriplex halimus* leaves under salt stress. Moreover, in *Casuarina glauca*, trehalose concentration significantly enhanced in roots at both 400 and 600 mM NaCl and simultaneously decreased carbohydrates such as fructose, sucrose, and glucose under salt stress [41, 42].

Vacuoles have a potential role in plant cell functioning. Salinity stress was the major challenge during the evolution of terrestrial plants [43]. SV (slowly activated vacuolar channels) are the most copious ion channels in vacuoles of plants, and these channels were the first discovered channels [44]. The crystallographic structure of SV channels has been published in 2016 from *Arabidopsis thaliana* [45, 46]. To prevent the salt from reaching the leaf surface, plants adopt two techniques. Salt ions either pass into the vacuole or get collocated in the apoplast. The amount of salt ions should not exceed the quantity accumulated in the vacuole [47].

Osmotic adjustment is a physiological adaptation of the plants that have drawn attention from past several years in response to salinity stress [13, 48]. Studies on physiological responses reveal that *Cakile maritima* species show responses to salinity stress through the mechanism of osmotic adjustment and selectivity of potassium ion over sodium ion [49, 50]. For maintaining the turgidity of the cell, osmotic adaptations play a significant role which enhances the productivity of the crop and development of the plant [19]. Contradictory findings about the role of proline in salinity tolerance have been reported. A markedly higher accumulation of proline took place in the leaf tissues of the salinity-sensitive cultivated than that of the wild barley genotype [40] and a nonsignificant contribution of proline in the salinity tolerance of *Aegilops cylindrica* species [51] observed at high levels of NaCl treatments. The puzzling differences in the literature regarding the contribution of proline to salinity tolerance can be explained by several facts: (1) the duration of the treatment, (2) the intensity of salinity stress, (3) the genetic background of the tested species, (4) the physiological stage of the sampling plants, and (5) the environmental conditions in which the plant grows [18]. In addition to proline contents, among the solutes, the soluble sugar content has an impressive role in the osmotic adjustment process under abiotic stresses. In melon (*Cucumis melo*) cultivars exposed to salinity stress, both proline content and total sugar content were significantly increased [52]. Osmotic adjustment in some plants is accomplished by consuming excessive levels of inorganic ions [35, 53, 54].

Salinity stress at moderate concentrations for long periods (months) or at high concentrations even in short periods (weeks) causes growth inhibition and ultimately death of the plant. Tolerance to salinity is attained through interconnected mechanisms [55]. In order to improve salinity tolerance in plants, ion and osmotic homeostasis play a significant role in stressful environmental conditions. The final decisive factor of these mechanisms includes several ion transporters which have a role in distributing toxic ions at cellular and organ levels [56]. Studies on salinity tolerance reveal an important issue to decide which transporters restrict or allow the sodium entry into the cell. A high accumulation of sodium ions in the cytoplasm restricts the enzymatic activities [57]. The vacuolar layer consists of two types of H^+^ pump namely V-PPase (vacuolar pyrophosphate) and V-ATPase (vacuolar type H^+^-ATPase) [58, 59]. These antiporters move the excess salt from the apoplast to the cytoplasm of the cell, and sodium moves to the vacuole in order to maintain osmotic regulation [60]. V-ATPase is the reigning H^+^ pump available in the plant cell. The stability of the plants is controlled by the activity of V-ATPase during stress conditions [35]. NHXs (Na^+^/H^+^ exchangers) sectionalized the Na^+^ into vacuoles. Na^+^/H^+^ antiporter is directing the partition of Na^+^ to the vacuole, thus limiting the fixation of Na^+^ in the cell cytoplasm. Activity of Na^+^/H^+^ antiporter is arbitrated by the effluence of Na^+^ from the roots of the plant which is encouraged by the SOS1 (salt overly sensitive) protein [61].

The concept of Na^+^/K^+^ discrimination, in which uptake of Na^+^ is exchanged by K^+^, allow the plant to tolerate the salinity stress. That is why the concept of Na^+^/K^+^ discrimination is taken into account as a significant basis in commercial crop selection. But this Na^+^/K^+^ discrimination concept is not a basis of salinity tolerance in glycophytes. In few salt-tolerant cultivars of barley and their wild relatives, there is not any trait for Na^+^/K^+^ discrimination [44]. Halophytes favor inclusion of Na^+^ over K^+^ which acts as a tool in osmotic adjustment and shows a positive correlation between salt tolerance and inclusion of Na^+^ in plants [45]. In cytosol, plant K^+^ requires approximately 100 mM of K^+^ ion for activities of cytosolic enzyme, whereas in vacuoles, K^2+^ ranges from 10 to 200 mM. The K^+^ ion transport from vacuolar tissues maintains cell turgidity through membrane channels and K^+^ transporter against the concentration gradient. In case of low K extracellular concentration, the affinity mechanism of K increases actively. As a result, K^+^ concentration in soil favours the uptake processes. Similarly, a decreased Na^+^ ion concentration (1 mM or less) is managed in the cytosolic region. Such enhanced level of Na^+^ concentration in soil under salt stress alleviates the competition between K and Na ions for similar mechanism causing diminishing of K^+^ uptake [42, 61].

Reduction in photosynthetic rate, stomatal/non-stomatal factors, deterioration in chlorophyll and carotenoid pigments, and chloroplast degradation were observed when exposed to salt stress as observed in *Phaseolus vulgaris*, *Zygophyllum xanthoxylum*, and *Lycopersicum esculentum* [62, 63]. Mutant studies revealed the role of ion transporters and channels in regulating chloroplast function during salt stress. The mutant lacking two chloroplast-localized K^+^ efflux anti-transporter (KEA1 and KEA2) demonstrates lowered photosynthetic efficiency, and exposure of Na^+^ in these mutants can improve phenotypic traits. However, chloroplasts give retrograde signals to connect chloroplast status, stimulating signalling cascade related to salinity response. In a nutshell, Na^+^ affects photosynthesis by degrading chloroplast function and proton motive force and by disrupting the function of Co_2_-functioning enzymes [61].

In halophytes, ion detoxification and osmo-protective strategies comprise Na^+^ extrusion from the roots to the xylem cell. The vacuolar compartmentation involving NHXs causes ion transportation. Several halophytes synthesize high Na^+^ in shoot than roots while maintaining increase in concentration of K^+^ as compared with glycophytes, and thus maintaining Na^+^/K^+^ ratio. The above findings confirm that halophytes employ various strategies in ion homeostasis and transportation in salinity stress condition. Similarly, recent genetic studies on the expression of *HKT1* and *SOS1* genes in *Eutrema parvula* (*EpHKT1:1*); *AcNHX1, AcSOS1,* and *AcHKT1* in *Aegilops cylindrica,* and *E. salsugineum* (*EsSOS1*) conferred higher salt tolerance as compared with other homologous analogous *AtHKT1:1* and *AtSOS1* in Arabidopsis [61, 64, 65]. Apart from salinity-exclusion mechanisms, halophytes have also evolved salt-avoidance mechanisms such as excretion (salt glands, bladder hairs, and re-translocation) and succulence for sodium dilution [66].

### Biochemical aspects of salinity tolerance

Plants engage different mechanisms to ensure salinity tolerance. At present, findings regarding the metabolic changes due to salinity tolerance are partial. A deep insight in metabolic as well as biochemical processes involving salinity tolerance is necessary for engineering of crop plants against salinity stress.

#### Heat shock proteins (HSPs)

HSPs are dispersed extensively in nature; also, these proteins pile up during stress conditions. Heat shock proteins are molecular chaperons which have a significant role in gathering and folding of proteins and eradication and destruction of non-useful proteins. Heat shock proteins are categorized in accordance to the molecular weight. These include small family Hsp, Hsp 100 family, Hsp 90 family, Hsp 70 family also known as DnaK family, and chaperonins such as Hsp60 and GroEL [67]. Salinity as well as drought stress is instigated by heat shock proteins (HSPs). Under stress conditions, several heat shock proteins are found to be upregulated such as HSP70-9-12 and -33 in poplar [68] and HSP70 in wheat [69] and rice seedlings [70]. Additionally, other heat shock proteins were upregulated in salinity stress such as HSP100-75 and -21; HSP90-12 and -9; HSP40-117 and -113; HSP60-49, -38, -33, and -31; and HSP21 in poplar [62] and HSP40 in rice [71]. Heat shock proteins such as HSP90 in Arabidopsis [22] and small HSPs, Clp (D1, D2), Clp (B1, B2), and HSP100 in rice crop [72, 73] exhibited salinity stress tolerance. Heat shock protein’s role in salinity stress is genotype specific in which HSPs were instigated more in cultivars which are salinity tolerant, and these were reported in soybean [23]. Studies revealed that heat shock proteins (HSPs) are significantly involved in tolerance to salinity stress. Transgenic tobacco with HSPs of *Medicago sativa* showed increased salt tolerance when compared with wild types at germination stage. However, overexpression of HSP-related transcription factors alleviated heat and enhanced the susceptibility to ABA and salinity stress in transgenic Arabidopsis. Moreover, increased expression of HSP genes in *H. vulgare* have been studied in site-specific salinity stress [53, 56]. According to Cen et al. [74], ion transport efficiency of a plasma membrane intrinsic protein gene namely *HvPIP2;8* was found when *Xenopus laevis* was studied. It has also been reported that the expression of *HvPIP2;8* significantly increased the movement of Na^+^/K^+^ salt as well as water transport, ultimately leading to salinity stress tolerance in *H. vulgare.*

Moreover, overexpression of Ipomoea batata Myo-inositol-1-phosphate synthase 1 (*IbMIPs1*) positively stimulates salt stress in transgenic plants. The contents of Na, H_2_O_2_, and melon dehydrogenase (MDA) directly reduced, whereas the phosphatidic acid, inositol, proline, trehalose, ABA, K^+^, and Ca^+^ levels significantly enhanced in transgenic *I. batata* under salinity stress [75]. In addition, introgression of the arginine decarboxylase gene into *Lotus tenuis* resulted to salinity tolerance by upregulating proline content, which maintains membrane integrity. However, transgenic *Triticum aestivum* containing the *Choline dehydrogenase* gene of *E. coli* were highly tolerant to salt stress due to enhanced Glycine betaine content in transgenic plants. Comparatively, *Gossypium hirsutum* contains *Choline monooxygenase* genes derived from *Atriplex sp*. enhancing the level of Glycine betaine in transgenic plants, resulting to membrane integrity under salinity stress [42].

HSPs are mainly confined in various cell organelles and play a key role in protein homeostasis, prevent protein folding, refolding of unfolded polypeptides, etc. when exposed to salinity stress. These proteins reduce production of ROS and prevent from oxidative damage in response to salt stress. It also prevents degradation of chloroplast structure and reduction of chlorophyll content and photosynthetic content. Specifically, HSP70 serves as an anti-apoptotic proteins and also induces programmed cell death in transgenic plants. The upregulation of HSPs helps in protection of photosynthetic machinery [76].

#### Small ubiquitin-like modifier (SUMO) protein

SUMO proteins come under the small protein family. Post-translational modification of the SUMO protein is SUMOylation which is having a crucial role in transcriptional regulation, stability of protein, various stress responses, apoptosis, and nuclear-cytosolic transport [77]. SUMO shows reversible nature in linkage onto substrate, and SUMO proteases have a crucial role in course of SUMOylation. OTS1 (overly tolerant to salt 1) and OTS2 (overly tolerant to salt 2) are the two SUMO protease which have been identified in the *Arabidopsis thaliana* to regulate responses to salinity stress [78]. The SUMO protein acts as a critical regulator in response to salinity stress in rice crop. The activity of SUMO protease has been demonstrated by the researchers for the orthologue *OsOTS1* and reveals that it plays a crucial role for tolerance to salinity in rice crop [79].

#### *Polyamines* (*PAs*)

PAs are omnipresent, tiny, polycationic aliphatic low molecular weight molecules copiously strew in the plant kingdom and have vigorous biological activity [80, 81]. Polyamines are found to play significant roles in normal growth, seed germination, and development such as cell proliferation regulation, morphogenesis, differentiation, breaking of dormancy, somatic embryogenesis, etc. [20, 82]. Most wonted polyamines are triamine spermidine (SPD), tetra-amine spermine (SPM), and diamine putrescine (PUT) [45, 83]. Out of these polyamines, diamine putrescine is the smallest one and is made from either ornithine or arginine [20, 84]. External application of polyamines (PAs) mainly SPD and SPM results in the enhancement of photosynthesis and reactive oxygen metabolism which ultimately improve the growth of the plant and alleviate the salt effect [85, 86]. Analogous results have also been attained in the seedling study of soybean crop [87]. Various metabolic pathways are found to be affected by the PAs namely SPD and SPM [88]. ABA and polyamines in combination helps in reducing the effect of salt in seedlings of grape [89].

The alleviated activities of phospholipase C (PLC) and phospholipase D (PLD) were observed in rice and Arabidopsis, when exposed to salt stress. Biosynthesis of PAs stimulates downstream regulation of Ca^2+^. The Ca^2+^-independent ζ-type PLD affects the movements of root growth toward salt-prone sites. Additionally, PA itself influences the regulation and movement of auxin and abscisic acid, two phytohormones induced during salt stress conditions. However, PAs also affect Na^2+^ transport via mitogen-activated protein kinase (MAPK6), that influences downstream regulation of *NHX7/SOS* [90]. In another study, rice MAPK6 regulates downstream activity of effector-like lectin RLK Salt I, Intolerance 1 and dephosphorylated through enzyme like protein phosphatase 2A and ultimately affect salt sensitivity and ethylene homeostasis. More precisely, PAs elicit activation and localization of stress-related proteins and osmolyte, thereby simulating Na^2+^ transport and hormone signalling [62, 91].

#### Antioxidants

Oxygen is a statutory constituent in the plants and is related with some processes like oxidative phosphorylation, metabolism, and mitochondrial respiration to provoke energy. In the course of the metabolic process, oxygen is transformed into ROS (reactive oxygen species). ROS constitutes hydroxyl radical, hydrogen peroxide, singlet oxygen, etc., and concentration of these ROS enhance during salt stress resulting in the cell death, irreversible metabolic dysfunction, and cytoplasmic membrane damage [92].

SOD (superoxide dismutase) has the ability to eliminate the large amount of superoxide anions from the cells and therefore acts as a defense system for salinity stress. These SODs are categorized into three classes. These classes are copper/zinc SOD (Cu/Zn-SOD), manganese SOD (Mn-SOD), and iron SOD (Fe-SOD). The superoxide anions can dismutase into the H_2_O_2_ and O_2_ with the help of SOD that eradicate the toxicity of superoxide [93]. CAT (catalase) is an enzyme which is present in peroxisomes and it deplete the amount of H_2_O_2_. High empathy for hydrogen peroxide makes it different from APX (ascorbate peroxidase), and it entails a reductive substrate. Catalase (CAT) eliminates the hydrogen peroxide (H_2_O_2_) which is formed by light reaction [94]. APX is an important enzyme which helps in the elimination of hydrogen peroxide. APX eliminates the hydrogen peroxide (H_2_O_2_) from the cell which is produced in the chloroplast by Miller reaction [95]. External application of ascorbate to different plant species helps to alleviate the inauspicious effect of salt stress [96, 97]. In Arabidopsis, salt stress–stimulating production of apoplastic reactive oxygen species (ROS) including hydroxyl radicals, superoxide, singlet oxygen, and hydrogen peroxide influences oxidative damage and disrupts redox potential. The expression of Respiratory Burst Oxidase Homologs (*RBOHs*) genes is stimulating dynamically, and they liberate salt-generated ROS waves. Under salt stress, these complicating ROS biosynthesis and production network are active constantly and play salt tolerance response [62]. Under salt stress, the amino acid concentration increased in two salt susceptible lines and three salt tolerance lines in *Oryza sativa*. Leucine, phenylalanine, isoleucine, and proline contents were enhanced among five lines. A recent study showed that the levels of α-aminobutyric acid, glycine, leucine, threonine, alanine, and serine glutamate reduced, whereas citrulline, ornithine, aspartic acid, cysteine, ornithine, valine and proline levels were significantly enhanced in *Cucumis sativa* when exposed to salinity stress [98].

Similarly, flavonoids including quercetin 3,30,7-tri-O-sulphate, cyanidine, quercetin, luteolin cyanidine 3-arabinoside chloride were significantly enhanced in soyabean roots in salt stress [99]. Metabolomics and quantitative phosphoproteomic studies revealed that MYB 17 optimizes flavonoid metabolism in *Glycine max* under salinity stress conditions. According to Xu et al. [100], activities of flavonoids associated such as flavonol synthase (*AvFLS*), flavanone 3-hydroxylase (*AvF3H*), and flavonoid 30-hydroxylase (*AvF30H*) genes were upregulated in *Apocynum venetum* seedlings under salt stress. Similarly, in *Zea mays*, cell wall content was reported to have decreased lignin, cellulose, and matrix polysaccharides in both shoot and root regions [101].

Dimethylsulfonium compounds contribute in the maintenance of protein integrity and ROS scavenging under salt stress. Glycine betaine as an osmoprotectant significantly improves proline content so effectively, and enhanced glutathione peroxidase and glutathione-S-transferase leads to the reduction of membrane peroxidase in different crop plants (rice and barley). Besides these, asparagine, sucrose, and glycine betaine contents significantly increased in maize shoots, whereas aspartic acid, malic acid, and γ-aminobutyric acid in roots when exposed to high salt stress [44, 102].

Recently, in *Solanum lycopersicon*, exposure of melatonin enhanced plant growth, carbohydrate content, and chlorophyll content and alleviated the enzymatic activities of ribulose-1,5 bisphosphates and carbonic anhydrase; these were recorded under salt stress. Additionally, melatonin exposure improves osmoregulation by alleviating activities of proline, soluble sugars, and other stress-dependant enzymes [62, 103].

According to Cen et al. [74], exogenous application of melatonin significantly improves H_2_O_2_ scavenging and increases antioxidant enzymes in *Medicago sativa* under salt stress conditions. Similar findings were reported by Quan et al. [104], that tolerant *M. sativa* exhibits reduction in accumulation of ROS as well as lower risk of membrane damage than salt-sensitive types. In another study, protein oxidation and lipid peroxidation occur in the apoplastic region when exposed to high salt condition. An enhancement in POX levels lowers H_2_O_2_ using different substrates, whereas activities of ascorbate peroxidase, catalase, and superoxide dismutase were increased in salt-tolerant *M. sativa* [105].

Kiani et al. [106] studied the antioxidant and protective role of polyphenol against salinity stress and the differential responses of genotypes using highly salt-tolerant, moderately salt-tolerant, and salt-sensitive genotypes. The vigorous antioxidant activity and robust accumulation of phenolic compounds in the leaves of the male parent (*Ae. cylindrica* Host) and amphidiploid derivates would imply greater sophistication in genetic diversity for the evolvement of defense-oriented strategies to prevent the accumulation of intracellular free radicals generated under salt stress.

### Gene expression and salinity

**Fig. 2:**
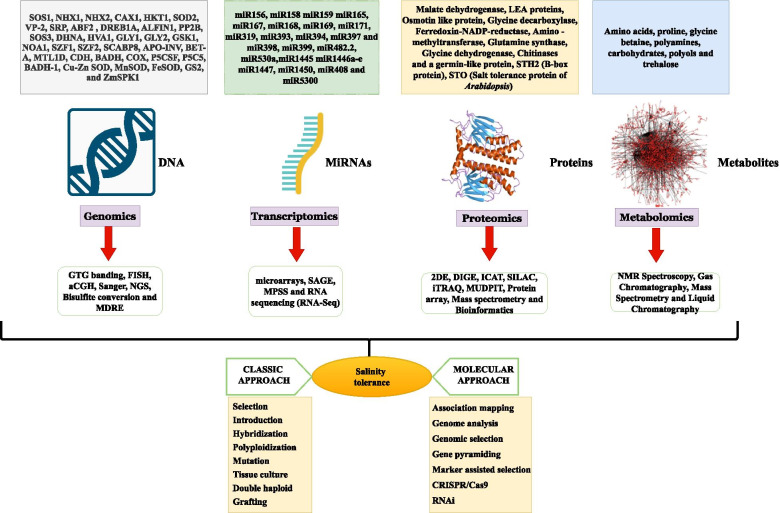
Diagrammatic representation of different “omics” approaches which are joined to each other at molecular level related with salinity stress tolerance in crop plant. *Abbreviations used:* SOS1 (Na^+^/H^+^ antiporter); NHX1 & NHX2 (Na^+^/H^+^ antiporter); CAX1 (cation/proton antiporter); HKT1, SOD2 (vacuolar Na^+^/H^+^ antiporter); VP-2 (vacuolar Na^+^/H^+^ antiporter); Srp (serine-rich protein); ABF2 (ABRE-binding bZIP transcription factor); DREB1A (transcription factor); ALFIN1 (zinc finger transcription factor); *PP2B* (signalling regulator); *SOS3* (calcium-binding protein); *PpDHNA* (dehydrin protein); *HVA1* (group 3 late embryogenesis abundant protein gene); *Gly1* and *Gly2* (glutathione-based detoxification of methyl glyoxal); *AtGSK1* (homologue of *GSKS3*/shaggy-like protein kinase); Atnoa1 (impaired nitric oxide synthesis); *AtSZF1* & *AtSZF2* (CCCH-type zinc finger protein); *SCABP8* (interacts with SOS2); Apo-Inv (apoplastic invertase); *bet* A (choline dehydrogenase), *mtl1D* (mannitol-1-phosphate dehydrogenase); CDH, BADH (glycine betaine synthesis); *Cod A* (glycine betaine synthesis); *COX* (choline oxidase (glycine betaine synthesis)); *Mtl1D* (mannitol-1-phosphate dehydrogenase); *p5csF* (proline synthesis); *mt1D* & *Gut D* (mannitol-1-phosphate dehydrogenase and glucitol-6-phosphate dehydrogenase); *P5C5* (pyrroline carboxylate synthase (proline synthesis)); BADH-1 (betaine aldehyde dehydrogenase); *Cu-Zn SOD* (copper zinc superoxide dismutase); *Mn SOD* (manganese superoxide dismutase); *Fe SOD* (iron superoxide dismutase); *GS2* (glutamine synthetase); *ZmSPK1* (sucrose non-fermenting-1-related protein kinase) malate dehydrogenase; LEA (late embryogenesis abundant proteins; *STH2* (B-box protein); STO (salt tolerance protein of Arabidopsis)

Molecular responses to abiotic stress consist of a number of genes and signalling cascades which are highly regulated and facilitate the crop plants to cope with the stress conditions. Regulation is mostly at transcriptional, post-transcriptional, and post-translation levels, but the main emphasis is at the transcriptional level which includes chromatin remodelling and upregulation and downregulation of the coding regions of the gene [107, 108]. Salinity tolerance is a composite and quantitative genetics process which is controlled by several number of genes [52, 109]. Several transcription factors as well as salt-responsive genes have been recognized with the help of genomic and transcriptomic approaches (Fig. 2). Among the gene families, it has been found that the SOS gene family plays a crucial role in response to salt stress in ion homeostasis [110].

Physiological responses to stress exhibit recent progresses in molecular work which show the detection of several genes having a role in salinity stress (Table 2). Transcription factors are assessed as principal regulators which have a key role in controlling the expression of genes. Dehydration responsive element binding (*DREB*), *NAC*, *APETALA2* (*AP2*), *C2H2*, *WRKY*, and *bZIP* (basic leucine zipper protein) families of transcription factors consist a huge number of members for stress-responsive genes. The *bZIP* gene expression has been observed by the scientist showing the upregulation of genes in wheat variety which is salt sensitive, but there is a downregulation of genes in the salt-tolerant variety of wheat [51]. The NAC family of transcription factors shows the overexpression of genes in wheat and rice crop resulting in the salinity tolerance by the plants thus have key role in alleviating the effect of stress. Some of the transcription factors are modulated by various kinases which have a crucial role in the adaptation of plants to salinity stress. It has been reported that transcription factor *OsRMC* (*Oryza sativa root meander curling*) in rice crop coding for receptor-like kinases which is explained as a pessimistic regulator for response to salt stress. It is also reported that the transcript level of gene *OsERBP1* is not influenced by salt, severe cold, or ABA significantly, but it is only regulated faintly by moderate cold and drought.

**Table 2:** Involvement of genes in functional aspects and mechanism of salinity tolerance

Sr. no.	Genes	Function during salt stress	Mechanism of action	References
1	*SOS1*	Transport of sodium ion from root to shoot of the plant	The protein of *SOS1* gene acts as antiporter of plasma membrane Na^+^/H^+^.	[111]
2	*SOS2*	Protein kinases	C terminal domain of *SOS2* associates with salt stress evoked Ca^2+^ via NAF domain (also called as FISL motif).	[112]
3	*SOS3*	Calcium-binding protein	SOS protein as well as Ca^2+^ behave as intonation of intracellular Na^+^ homeostasis.	[113]
4	*ERF1 (SERF1)*	Improve salinity tolerance	*SERF1* gene attaches with the promoter region of MAP 3K6, MAPK5 to show tolerance against salinity stress.	[114]
5	*HVP1 and HVP10*	Expressed during salinity stress	These two genes express itself in the presence of ABA in *Hordeum vulgare.*	[115]
6	*TIP1 and GLP1*	Expressed upon salt stress	Treatment of ABA on the wheat plant shows the expression of these genes against salt stress.	[116]
7	*rd29A*	Act with *DREB2A* transcription factor	This transcription factor *DREB2A* is induced by salt stress in the Arabidopsis plant.	[117]
8	*TaWRKY2, TaWRKY19*	Improved salt tolerance	Overexpression of these genes improve salt tolerance by enhancing the downward expression of genes *RD29B* and *STZ.*	[118]
9	*OsCLCa*	Decrease in the salt concentration in the rice plant	This gene acts through the leaves and roots of the plant, and there is decrease in the transcript accumulation in the variety of rice IR29 which is salt sensitive.	[119]

Abbreviations used: *SOS1* salt overly sensitive 1, *SOS2* salt overly sensitive 2, *SOS3* salt overly sensitive 3, *ETF1* ethylene response factor1, *HVP* hordium vulgare vacuolar H+ -pyrophosphatase, *GLP-1* glucagon-like peptide-1, *TIP-1* tip elongation protein 1, *Rd29A* response-to-dehydration 29A, *OsCLCa* Oryza sative chloride channel-a, *TaWRKY* transgenic tobacco WRKY

### Breeding strategies

Many researchers have been studying breeding for tolerance to salt [120–122]. There have been many investigations in screening and breeding for salinity tolerance in crop plants, and the subject has been reviewed by several authors [123–126]. With the advancement in genetic inheritance, evaluation techniques, software techniques, molecular markers, modification of germplasm and mapping, it facilitates the improvement in salt tolerance and other abiotic stresses [127]. Majority of the plant processes which are having a role in salinity tolerance show continuous diversity, have a little inheritance, and are also affected by environmental factors [26, 121].

#### Mutation breeding

Mutation breeding, a tool for creating genetic variation, is useful for crop improvement. A key point in mutation breeding is identification of individuals with a target mutation involving two major steps: screening and confirmation of mutants [128]. In mutation breeding, the seeds are treated with mutagen (agents such as gamma rays, chemical mutagens, X-rays, and fast neutrons) and grown further for segregation and the plants with useful traits are selected to grow next generation. Multi-location trials are conducted for evaluation and released as a new variety with use [129]. Studies have been done to improve the salinity tolerance in plants. There is an evidence of salt oversensitivity which is achieved through mutation breeding in barley crop that show improvement in salinity tolerance through ion homeostasis. There is an interpretative difference between the salt-tolerant genotypes of barley (i.e., M4-73-30 and its wild type cv). These two genotypes show the difference in the expression of *HVA* (202-fold), *HvSOS3* (31-fold), *HvSOS2* (24-fold), and *HvSOS1* (105-fold) genes in the roots of barley. There is more Na^+^ accumulation in wild-type barley sample of shoot than the mutant type. This is because of the less transfer of sodium ions from root to shoot in the mutant type which is salt tolerant [130]. Lethin et al. [131] developed a mutant population of wheat with the aim to improve salinity tolerance in wheat. They used Bangladeshi variety BARI Gom-25 which was semi-tolerant to salinity and treating it with EMS (ethyl methanesulfonate) and then compared it with local wheat varieties. After screening, they identified that out of 1676 lines of wheat, 70 lines manifested enhanced salinity tolerance. Results indicated that the mutant lines showed a good salinity tolerance than the local cultivars [131].

#### Wild relative exploitation

Interspecific hybridization has a crucial role in ameliorating the crop plant performance for tolerance to several abiotic stresses [132, 133]. Wild relatives of crops are utilized as a source in biotic as well abiotic stress tolerance to increase the crop productivity; however, it requires specialized methods to do so, such as embryo rescue. The salt tolerance source identification as well as the involvement of a candidate gene is a good example found in wild rice germplasm [134–136]. *Oryza coarctata,* which is a halophytic relative of wild rice, has been studied for tolerance to salt for decades for identification of a way in which it can be utilized in salt tolerance improvement of cultivated rice [136, 137]. Colmer et al. [132] have reappraised the scenarios for salt tolerance improvement in wheat crop by using wild relatives of wheat. Genetic and physical maps as well as genome sequences of wild relatives of some crops are becoming accessible [138, 139].

#### Double haploid (DH)

Double-haploid production through Anther culture has emerged as an exciting tool for crop improvement having advantages of shortening of the breeding cycle, high selection efficiency, homozygosity fixation, and expression of recessive alleles suitable for breeding. Diploidization of haploid genomes can be produced either by artificial genome doubling (colchicine treatment) or spontaneous genome doubling (endomitosis: chromosome doubling without nucleus division). DH technology was found to be efficient for fixation of favorable alleles controlling agronomically important traits. In rice crop, DH techniques can be utilized for the development of new varieties from photosensitive rice genotypes [140]. Double haploids could emerge as a powerful tool in mapping of QTLs controlling quantitative traits. QTLs linked with sheath blight of rice were identified in a DH population exhibiting resistance against the disease. More than 100 rice breeding varieties have been developed in India, South Korea, the USA, China, and Japan [141]. DH technique produces the homozygous lines of haploid plants by chromosome doubling from sperms or egg cells. Several reviews on DH technology provide an insight to the plant breeders in crop improvement as it has wider application in breeding and genetic study [142, 143]. Al-Ashkar et al. [7] detected salt tolerance of 15 lines of wheat which was developed using DH technique. They analyzed the biochemical as well as physiological parameters and then compared it with wheat check cultivar sakha 93 which is salt tolerant [7].

#### Marker-assisted breeding (MAB)

The marker-based selection is an indirect selection process in which the trait of interest is selected on marker basis rather than phenotypic selection. It is the application of molecular biotechnologies, generally molecular markers combined with linkage maps and genomics for the improvement of plant and animal traits that are based on genotypic assays. By increasing the number of markers associated with QTL, could greatly increase the success rate [144, 145]. For efficient marker selection, marker to be used should be close enough to the gene of interest. Molecular markers have become an integral part of the plant breeding and classical genetics. The main reason behind it is that molecular markers made it possible to do selections and breeding for any trait. Earlier, development of molecular markers, linkage map construction, QTL mapping, and fine mapping of precise gene were considered to be labor-intensive and time-consuming processes. But with the advancement of next-generation sequencing (NGS), it has made the development of molecular markers, like simple sequence repeats (SSR), insertion-deletions (InDels), and single-nucleotide polymorphisms (SNPs) easier. The development of these markers has further facilitated the development of high-density genetic maps, which in turn enabled the mapping of target genes. The detection of genetic variation for important agronomic traits is also done using molecular markers. Molecular markers enabled the identification of appropriate parents for molecular breeding and also made it possible to select the desirable offspring at the early developmental stages [146, 147]. To alleviate the effect of salt on production of rice, new varieties which are salt tolerant are developed using marker-assisted breeding [148–150] and conventional breeding methods [151–153]. MAB is a rapid and accurate method of breeding for introgression of lines or genes. It allocates selection in every breeding cycle for the transfer of gene in a precise manner. MAB also permits restricting the donor region thereby eluding linkage drag [148]. Marker-assisted breeding (MAB) was fortunate in the development of salinity tolerance lines in rice [148, 149, 154–158]. In rice, marker-assisted breeding has been utilized in pyramiding of QTLs which control tolerance against salinity, submergence, as well as drought [6]. Pushpavalli et al. [159] studied the chickpea genotypes for salinity tolerance and identified two yield-related QTLs in the key genomic region. Comparison with already published chickpea genetic maps showed that these regions conferred salinity tolerance across two other populations, and the markers can be deployed for enhancing salinity tolerance in chickpea.

### Genetic engineering for salt tolerance in plants

Salinity tolerance is predominantly supervised by numerous genes as well as various physiological mechanisms. Genes which are related to salt tolerance provides a presumed postulation of the stress signal network for amplification and enhancing the tolerance of plants to various stresses [160]. The technology of genetic transformation facilitates scientists to attain the transfer of gene in an anticipatory and accurate manner. That is why, scientists’ concern is on the plant transformation in order to improve the salt tolerance by operating the osmoprotectant biosynthetic channel for the accumulation of molecules which perform by narrowing the lipid peroxidation, function and structure of protein, ROS scavenging, etc. [161, 162]. Studies on gene expression by employing constitutive promoters furnish restricted biological information as compared with the use of cell type-specific promoters or inducible promoters. That is why engineering for salt-tolerant crops could be done by miRNA overexpression, using synthetic biology basis in order to enhance strategies for genetic engineering, maintenance of hormone homeostasis to eschew pleiotropic effects, complete knowledge of post-translational modifications, fortunate fine-tuning of response to stress by engineering innovative regulatory targets [163]. Genes which are used for genetic engineering of salt-tolerant crops include water channel proteins, detoxifying genes, dehydrins, osmoprotectants, ion transporter and molecular chaperons (Table 3). In transgenic plants, S-adenosylmethionine decarboxylase (SAMSC) plays an important role in the biosynthesis of PA, and its activities are reported to be enhanced under different salt treatments. Ectopic expression of *SAMDC*-like genes in rice enhanced the level of spm and spd that increased salt tolerance. In addition, the *OstA* and *OtsB*, two bifunctional fusion genes derived from *E. coli* in *O. sativa* are reported to increase trehalose content, amino acids under salt stress. According to Li et al. [91], the overexpression of *Oryza sativa* trehalose phosphate synthase (*OsTPS1*) shows increased trehalose synthesis and improved salt tolerance in transgenic plants. Similarly, proline and trehalose content were enhanced and upregulation of stress-related genes (responsive to ABA (*RAB16C*), early light-inducible protein (*ELIP*), water stress-inducible protein (*WSI18*), and heat shock protein (*HSP70*), in transgenic rice.

**Table 3:** Involvement of genes obtained from different crops and their role in stress tolerance

Sr. no.	Gene	Source of gene	Product of gene	Target plant	Effects	References
1	*ABP9*	*Zea mays*	Transcription factor	Arabidopsis	Salt, cold and drought tolerance	[164]
2	*TaMYB2A*	Triticum aestivum	Transcription factor	Arabidopsis	Salt, cold and drought tolerance	[165]
3	*TaSRG*	Triticum aestivum	Transcription factor	Arabidopsis	Salt tolerance	[166]
4	*ThNHX1*	*Thellungiella halophila*	Na^+^/H^+^ antiporter	Arabidopsis	Salt tolerance	[167]
5	*SsNHX2*	*Suaeda salsa*	Na^+^/H^+^ antiporter	Arabidopsis	Salt tolerance	[168]
6	*PutHKT2,1*	*Puccinellia tenuiflora*	K^+^ transporter	Arabidopsis	Salt tolerance	[169]
7	*ZmSIMK1*	*Zea Mays*	Mitogen-activated protein kinase (MAPK)	Arabidopsis	Salt tolerance	[170]
8	*HvCBF4*	*Hordeum vulgare*	CBF transcription factor	Rice	Increased drought, cold and salinity tolerance	[171]
9	*OSNAC5*	*Oryza sativa*	Transcription factor	Rice	Salt tolerance	[172]
10	*CgNHX1*	*Chenopodium glaucum*	Vacuolar Na^+^/H^+^ exchanger	Rice	Increased salt tolerance	[173]
11	*PgNHX1*	*Pennisetum glaucum*	Vacuolar Na^+^/H^+^ antiporter	Rice	Enhanced salt tolerance	[174]
12	*OsKAT1*	*Oryza sativa*	Shaker family K^+^ channel	Rice	Salt tolerance	[175]
13	*OsBADH*	*Oryza sativa (Indica)*	Betaine aldehyde dehydrogenase	Rice (*Japonica*)	Enhanced salt tolerance	[176]
14	*PcINO1*	*Porteresia coarctata*	Myo-inositol 1-phosphate synthase	Rice, brassica	Salt tolerance	[177]
15	*MIPS*	*Spartina alterniflora*	Myo-inositol 1-phosphate synthase	Rice, tobacco	Improved salt tolerance	[178]
16	*AtNHX1*	*Arabidopsis thaliana*	Na^+^/H^+^ antiporter	Maize	Salt tolerance	[179]
17	*BADH*	*Suaeda liaotungensis*	Betaine aldehyde dehydrogenase	Maize	Salinity tolerance	[175]
18	*H*^*+*^*-PPase*	*Thellungiella halophila*	H(+)-pyrophosphatase	Cotton	Salt tolerance	[180]
19	*SbGSTU*	*Salicornia brachiata*	Glutathione S-transferase	Tobacco	Increased salt tolerance	[181]
20	*AtNHX1*	*Arabidopsis*	Vacuolar Na^+^/H^+^ antiporter	Groundnut	Enhanced drought and salt tolerance	[182]
21	*ThIPK2*	*Thellungiella halophila*	Inositol poly-phosphate kinase	*Brassica napus*	Increased abiotic stress tolerance	[183]
22	*PcSrp*	*Porteresia coarctata*	Serine-rich-protein	Finger millet	Improved salt tolerance	[184]

### Novel Approaches in improving salt tolerance

#### CRISPR/Cas9 technique

CRISPR/Cas9 is an important gene editing tool in which the cas9 protein along with guide RNA from a complex for recognition of target sequences. In the system, the target DNA is cleaved by the Cas9 protein which consists of six domains namely REC1, REC2, Bridge Helix, HNH, RuvC, and PAM interacting. The Rec1 domain helps in binding the guide RNA, whereas the bridge helix (arginine rich) initiates the cleavage after binding of target DNA. The cas9 protein only activates when bind with guide RNA. The guide RNA is mainly composed of single-stranded RNA with 1 tetraloop and 2 or 3 stem loops, and it must have a 5’ for complimentary with target DNA sequence. The cas9 protein searches for target DNA for PAM sequences. The protein melts the upstream bases of the PAM and pair with them. In the case of exact target sequence, RuvC and HNH nuclease play a role to cut the target DNA sequence which then followed by the Watson-crick pairing between the DNA cas9sgRNA complex and guiding sequence [185, 186]. CRISPR/Cas9 is a precise, systematic, and appropriate method of genome editing which was developed recently [187]. Nowadays, the CRISPR/cas9 technique has been utilized extensively in several crops like maize [188, 189], wheat [190, 191], and sorghum [192, 193]. With the help of the CRISPR/Cas9 technology, numerous genes in rice crop such as *OsHAK1*, *OsERF922*, *OsPDS*, *TMS5*, and *Badh2* have been knocked out, and predicted results of phenotype have been obtained [194–197]. Some studies on the elite rice show direct genome editing of cultivar using the CRISPR/Cas9 technique. The gene *OsERF922* which is an ERF transcription factor was mutated by this technique to increase the blast resistance in variety Kuiku131 having normal phenotype [190]. Japonica rice cultivar WPB106 was resistant to drought, having good cooking quality and early maturity but sensitive to salt. Its tolerance to salt has been improved by using CRISPR/cas9 technology where they used the *Cas9-OsRR22-gRNA* expression vector which knocks out the *OsRR22* gene. Their results based on this technology illustrated that *OsRR22* has an auspicious prospective to advance the amelioration of salinity tolerance in rice breeding [198]. In a study in upland cotton (*Gossypium hirsutum*) a CRISPR/Cas9-mediated pooled sgRNA assembly was optimized providing a platform in sgRNA designing for targeting the multiple genes. The targeted genes were successfully edited using CRISPR/Cas 9 technique which were related to male sterility in cotton. A total of 112 plant development-related genes were knocked out using this system [199]. Chen et al. [200] successfully generated the first report of generating high-oleic and nontransgenic mutant in allotetraploid upland cotton by knockout of *GhFAD2* genes through CRISPR/Cas 9 editing system. Findings in upland cotton suggested that *GhFAD2-1A/D* is the key gene which determines the fatty acid composition of cottonseed oil.

#### Hyperspectral imaging (HI)

Also called imaging spectroscopy, it tells us about how the light interrelate with the materials which measure the quantity of light transmitted, reflected, or emitted [201]. Nowadays, various works emphasize on environmental stress analysis in crops and its related diseases [202, 203]. Hyperspectral imaging is a new technique used to evaluate tolerance to salt stress in wheat crop. There are three methods namely NRD (normalized reflectance difference) curve, posterior stability, and MDPA (minimum difference of pair assignments) which were used to scrutinize hyperspectral images of the lines of wheat crop. They used the four lines of wheat crop namely Kharchia, CS, Co (CS), and Sp (CS). It was concluded that among the four lines, kharchia is more tolerant to salt than the others [204]. HI is a technique used for the identification of material via imaging system providing high spectral resolution when compared with multispectral system, namely Landsat multispectral scanners. This system provides an insight for the improved identification of surface materials especially minerals in soils. HI system is based on the principles of red–green–blue (RGB) image in which an image is presented as a matrix with I rows and J columns providing the I*J dimensions which determine the size of the image. In this system, pixels are fact point measurements, instead of squares, in which each entry in the matrix represents with one pixel [205]. A pixel in an image represents the real space position which is absorbing and reflecting a light across the electromagnetic spectrum. In this system, the reflected light is counted as a number which indicates the intensity. The low intensity of the wavelength is represented by the black image, while high intensity with white and one wavelength showed greyscale image. The color bands lie within the electromagnetic spectrum of light (400–800 nm) which corresponds to blue, green, and red visible lights [206].

#### Genome-wide association studies (GWAS)

GWAS, an important tool, provides an insight in the identification of genotype–phenotype association and is mainly focused on linkage disequilibrium and recombination, and singlefeature polymorphism. In over 1000 GWAS studies on recombination and linkage disequilibrium [207], single-feature polymorphism [208], pathogen resistance, and early flowering [209] have now been published in plants [210, 211]. GWAS analysis includes the collection of data based on genotypic and phenotypic information in which genotypic data can be collected with the help of microarrays or whole genome sequencing (WGS) or whole exome sequencing (WES). Further, quality control is a major step in the analysis for the deletion of bad single-nucleotide polymorphisms (SNPs) followed by imputation using matched reference populations from repositories. Genetic association tests are conducted for each trait using various models (linear or logistic regression). In silico analysis of GWAS is carried out for in silico fine-mapping, gene to function mapping, genetic correlation, pathway analysis, and SNP to gene mapping [212]. In a study conducted on *Oryza sativa* using GWAS mapping revealed the novel QTLs at the seedling stage for salinity stress tolerance. GWAS analysis identified 26 QTLs after screening of 179 rice landraces genotyped with 21,623 SNP markers for salinity stress tolerance when treated with 100 mM NaCl treatment. From the identified QTLs, 10 QTLs were found to be associated with different traits [213]. QTL [214] analyses generally point to particular chromosomal subregions, while the current developed GWASs can recognize accurate location of chromosomes with the intention to elucidate particular genes or polymorphism within the encoding regions [215, 216]. GWAS studies divulge the candidate genes which underlie characters namely flowering time [217], morphology of root [218], size, shape and length of grain [216], and yield [219] in rice crop. Few studies are there which have applied the technique of GWAS to elucidate the molecular mechanism which induces tolerance [220]. GWAS analyses deliver light on phenotype and protein function when employing suitable population and genotyping of high resolution [8, 221]. GWAS is used to recognize markers for salt tolerance in rice crop. In this study, they apply GWAS to a diversity which show rice accessions throughout the globe, and these accessions show genetic variability in a high degree. A total of 950 genes were recognized belonging to several functional categories. These genes were overrepresented in Gene ontology (GO) classification of transcription regulation, hydrolase activity, and cation transport [222, 223].

### Conclusive remark and future prospects

On the basis of the physiological, biochemical, as well as molecular aspects, salinity stress tolerance has been extensively studied. Recent studies mainly focused on the molecular basis which acquires more scrutiny. The gene’s identification furnishes information regarding the mechanisms which are directly influenced by extracellular cues. Studies on salinity tolerance elucidated that several salinity responses, osmotic regulation, antioxidant metabolism, hormone metabolism, and signalling pathways play a crucial role in stress tolerance. Moreover, new emerging approaches of plant breeding and biotechnologies such as GWAS, mutational breeding, marker-assisted breeding (MAB), double haploid production (DH), hyperspectral imaging (HI), and CRISPER/Cas, serve as engineering tools for dissecting the mechanism in more depth. However, understanding of these mechanisms creates a loop of concept for breeders with more focus on plant performance under saline conditions. Moreover, pathways and routes in relation to salinity unravel different components that give exciting outcome to engineer plants for the search of novel salinity resistance genes.

## Data Availability

NA
